# Mobility and subcellular localization of endogenous, gene-edited Tau differs from that of over-expressed human wild-type and P301L mutant Tau

**DOI:** 10.1038/srep29074

**Published:** 2016-07-05

**Authors:** Di Xia, Julia M. Gutmann, Jürgen Götz

**Affiliations:** 1 Clem Jones Centre for Ageing Dementia Research, Queensland Brain Institute, The University of Queensland, St Lucia Campus, Brisbane, QLD 4072, Australia

## Abstract

Alzheimer’s disease (AD) and a subset of frontotemporal dementia termed FTLD-Tau are characterized by a massive, yet incompletely characterized and understood redistribution of Tau. To establish a framework for understanding this pathology, we used the genome-editing tool TALEN and generated Tau-mEOS2 knock-in mice to determine the mobility and subcellular localization of endogenous Tau in hippocampal cultures. We analysed Tau in axons, dendrites and spines at three stages of maturation using live-cell imaging, photo-conversion and FRAP assays. Tau-mEOS2 cultures were compared with those over-expressing EGFP-tagged forms of human wild-type (hWT-Tau) and P301L mutant Tau (hP301L-Tau), modelling Tau accumulation in AD and FTLD-Tau, respectively. In developing neurons, Tau-mEOS2 followed a proximo-distal gradient in axons and a subcellular distribution similar to that of endogenous Tau in neurons obtained from wild-type mice, which were abolished, when either hWT-Tau or hP301L-Tau was over-expressed. For the three conditions, FRAP analysis revealed a similar mobility in dendrites compared with axons; however, Tau-mEOS2 was less mobile than hWT-Tau and hP301L-Tau and the mobile fraction was smaller, possibly reflecting less efficient microtubule binding of Tau when over-expressed. Together, our study presents Tau-mEOS2 mice as a novel tool for the study of Tau in a physiological and a pathological context.

Tau is a microtubule-associated protein that is enriched in neurons, where it is expressed as multiple isoforms. In the human central nervous system, there are six low molecular-weight isoforms that are generated by alternative splicing^1^,^2^,^3^. This results in isoforms that have either 0, 1 or 2 N-terminal inserts (0N, 1N and 2N) and either three (3R) or four (4R) microtubule binding domains^4^. Tau is developmentally regulated in humans, with the foetal isoform corresponding to the shortest of the six adult isoforms^5^,^6^. Mice, in contrast, express only 4R isoforms in adulthood whereas in the foetal brain the major isoform is 3R0N^7^,^8^.

A major interest in Tau stems from the fact that this protein forms insoluble aggregates known as neurofibrillary tangles (NFTs) in many neurodegenerative disorders that are collectively termed tauopathies. These disorders include Alzheimer’s disease (AD), which is also histopathologically characterized by plaques that are composed of aggregated, fibrillar forms of the peptide amyloid-β (Aβ)^9^. Tau pathology in the absence of an overt Aβ pathology is characteristic of a subset of frontotemporal dementias (FTD; also termed frontotemporal lobar degeneration, FTLD-Tau^10^). Not surprisingly, many studies of Tau function use over-expression systems, both in cell culture and in animal models, in order to better understand how Tau (with or without FTLD mutations) causes neurodegeneration and ultimately dementia. One of the more prevalent FTLD mutations is P301L that was one of the earliest pathogenic mutations to be identified in FTLD^11^,^12^,^13^. Mechanistically it has been shown that the mutations reduce microtubule binding and facilitate Tau polymerization^14^,^15^; other mutations have been shown to affect the association of Tau with the plasma membrane^16^. Many wild-type (WT) and FTLD mutant forms of Tau have been expressed in mice, with transgenic expression of human P301L Tau facilitating NFT formation at an early age, different from human WT Tau-expressing mice where NFT formation is delayed^17^. These animal studies have been complemented by other investigations in cell lines which have shown, for example, that transient expression of WT or disease-linked mutations of Tau (R406W, P301L, ΔN296) had a dramatic effect on the microtubule cytoskeleton as observed by immunofluorescence microscopy^18^. Additional insight has been gained in the roundworm *C. elegans* where the Tau homologue Ptl-1 has been shown to regulate structural neuronal integrity^19^,^20^.

*In vivo*, Tau has been reported to be an axonal protein under physiological conditions, although a role has also been demonstrated in the dendritic compartment where levels are considerably lower^21^,^22^. In neuronal cultures, Tau has been shown to be ubiquitously expressed, with an enrichment being reported in the axonal compartment^23^. In tauopathies, Tau accumulates in the somatodendritic domain. Aβ incubation and synaptic activation have both been demonstrated to cause the accumulation of Tau in spines24. On the other hand, P301L Tau has been shown to accumulate in an Aβ-dependent manner in spines *in vivo*^21^. Similarly, over-expression of P301L Tau was also found to cause increased spine localization^25^. Together, these findings demonstrate that Tau localization to spines is tightly regulated.

In order to determine how Tau accumulation impairs neuronal function, it is important to understand how the protein is distributed in neurons, how it is localized, and the nature of its mobility characteristics. A few studies have assessed the transport of Tau under both physiological and pathological conditions, leading to the claim that, at least in axons, Tau is transported anterogradely through a combination of free diffusion and transient association with microtubules, hopping from microtubule to microtubule (‘kiss and hop’), with a reported 40 ms dwell time^26^, and diffusing along the microtubule lattice^27^. Some studies assessing Tau transport have also applied newer microscopy methods such as FRAP (Fluorescence Recovery After Photobleaching) or sptPALM (single particle tracking Photo-Activated Localization Microscopy)^28^ to over-expression systems^29^. Here, we generated a novel mouse strain using the genome editing tool TALEN to equip endogenous Tau (with its multiple isoforms) with an mEOS2 tag that could be used to track Tau and conduct photo-conversion. We then assessed the behaviour of the tagged Tau and compared it to that of over-expressed human WT (hWT-Tau) and P301L mutant Tau (hP301L-Tau).

## Results

### Tau-mEOS2 knock-in mice generated with the genome-editing tool TALEN express all three major central nervous system Tau isoforms

**Figure 1:**
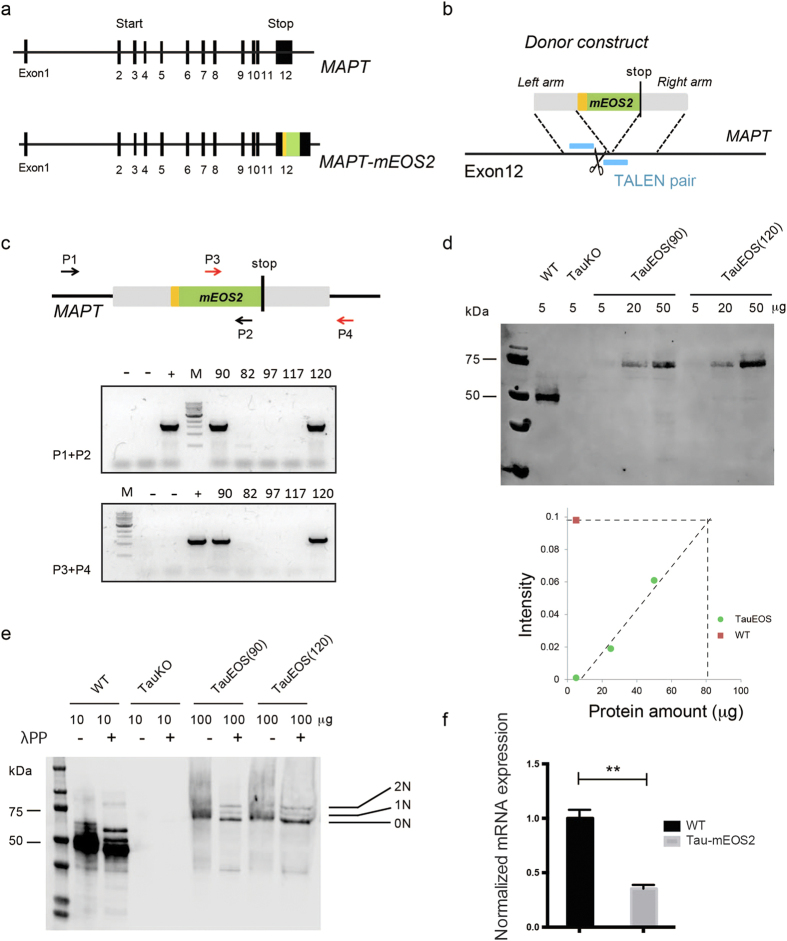
Generation of Tau-mEOS2 knock-in mice using the genome-editing tool TALEN. **(a)** Tau-mEOS2 knock-in strategy. Upper panel, murine *MAPT* locus. Lower panel, mEOS2 cassette (in green) inserted in-frame down-stream of *MAPT* exon 12 using a linker sequence (in yellow). **(b)** Homologous recombination of the donor construct into the target site using TALENs. A TALEN pair (in blue) targeting a sequence near the stop codon in exon 12 of the *MAPT* gene. The donor construct comprises a sequence encoding the photo-convertible fluorescent protein mEOS2 flanked by 800 bp each of the homologous arms. **(c)** Flanking PCR using two primer pairs (P1/P2, P3/P4) to identify mice that have integrated the donor construct by homologous recombination (HR) (Tau-mEOS2 mice) identifies two positive founders (#90 and 120) in 120 animals obtained by micro-injection. **(d)** Western blot analysis shows that the size of Tau-mEOS2 is about 75 kDa, which is approximately 25 kDa larger than endogenous Tau as predicted, indicating correct targeting of mEOS2 into the MAPT locus. Wild-type (WT) and Tau knock-out (KO) samples were included as controls. (**e**) Dephosphorylation reveals three murine central nervous system isoforms in Tau-mEOS2 mice with a 2N:1N:0N ratio similar to that in WT mice. **(f)** qRT-PCR analysis reveals almost three-fold reduced levels of *Tau-mEOS2* compared to WT *Tau* (two-tailed unpaired Students t-test, **P < 0.01, n = 3 per group).

The protein Tau is encoded by the *MAPT* gene, and alternative splicing generates multiple isoforms^3^. To visualize all forms of endogenous Tau *in vivo*, we established a novel transgenic mouse line by introducing a photo-convertible mEOS2 tag in-frame into the carboxy-terminus of the *MAPT* gene, using the genome-editing tool TALEN^30^ (Fig. 1a). Three TALEN pairs predicted to yield a high targeting efficiency and to have low off-target effects were designed and assembled using the TALEN toolkit provided by the Zhang Laboratory (Addgene Kit # 1000000019). A donor construct comprising the mEOS2 cassette flanked by 800 bp long arms both upstream and downstream of the genomic sequence containing the *MAPT* stop codon was designed (Fig. 1b). The TALEN reaction was established in N2A cells by evaluating three TALEN pairs for genome-editing activity using the T7 endonuclease I assay (data not shown). The pair with the highest activity was used for *in vitro* transcription, and the thereby generated TALEN mRNAs were combined with the donor construct and injected into pronuclei of C57Bl/6 single cell mouse embryos by classical transgenesis^31^, followed by transfer into pseudo-pregnant foster mice. To identify animals among the offspring (F0) that had undergone an mEOS2 targeting event, flanking PCR genotyping was performed, using two primer pairs (P1/P2 and P3/P4), with one primer binding to the mEOS2 sequence (P3, P4), and the other flanking primer binding to regions outside the donor construct sequence, one upstream of the left arm (P1) and the other downstream of the right arm (P4) (Fig. 1c). Of a total of 120 offspring, two mice (#90 and #120) were identified as positive. To ensure that the recombination had not induced INDEL (insertion or deletion) mutations, we PCR-amplified the top 5 predicted off-target sites (using the above software) and conducted Sanger sequencing; this confirmed the absence of mutations (data not shown).

We next established two homozygous Tau-mEOS2 mouse lines (TauEOS90 and TauEOS120) employing a sequential breeding procedure. We performed an initial analysis that included western blotting and the establishment of primary neuronal cultures with both strains, and found no overt differences between them. Subsequently, we only used the TauEOS90 strain, which is referred to as Tau-mEOS2 from here on. Western blot analysis using a pan-Tau antibody (DAKO Tau) and including WT and Tau knock-out samples^32^ as controls confirmed that the size of Tau-mEOS2 was about 75 kDa. As predicted, this is approximately 25 kDa larger than endogenous Tau, indicating correct targeting of the mEOS2 cassette (Fig. 1d). Tau is an unusual protein in that its longest human brain isoform contains 80 serine and threonine residues and 5 tyrosine residues, many of which are phosphorylated under physiological conditions. Owing to its basic amino acid content, its heterogeneous phosphorylation and the existence of multiple isoforms, the protein runs like a smear, and the individual isoforms can only be resolved by treating the extract with protein phosphatase^3^,^33^. By incubation with lambda phosphatase, the three murine central nervous system isoforms were revealed with a 0N:1N:2N ratio similar to that in WT mice, indicating that alternative splicing is maintained in the Tau-mEOS2 mice; the ratio in WT mice was 81:8.5:10.5, and the ratio in the Tau-mEOS2 mice was 70.5:8:21.5 (Fig. 1e). By titrating Tau-mEOS2 against WT extracts, we also found that the level of Tau expression was approximately fifteen-fold less than that of WT (Fig. 1d). To determine whether this was a reflection of reduced transcript levels, we used qRT-PCR and found an almost three-fold reduction in the levels of *Tau-mEOS2* compared to *WT Tau*, suggesting that the tag interferes with either the transcription or turnover of *Tau-mEOS2* mRNA (Fig. 1f). The Tau-mEOS2 mice, upon gross analysis, did not display any impairments. Together, these findings demonstrate that the Tau-mEOS2 strain is overtly normal, despite significantly reduced Tau levels, presenting it as a tool for the analysis of Tau mobility and subcellular localization in response to both internal and external stimuli.

### Over-expression of WT and P301L mutant Tau reverses the axo-dendritic gradient and abolishes the proximo-distal gradient in the axons of Tau-mEOS2 hippocampal cultures

Tau is conventionally treated as an axonal protein; however, we and others have previously demonstrated that, even under physiological conditions, Tau is also present in the dendrites and in spines, albeit at lower levels^21^. Tau has been further localized to neuronal nuclei^34^; it has also been found in nuclear fractions, in isolated intact nuclei and as speckles in the nuclei of SH-SY5Y but not N2a neuroblastoma cells^35^. These analyses used Tau-specific antibodies and, as far as conventional immunohistochemistry is concerned, required antigen retrieval methods, unless Tau was over-expressed as is the case for Tau transgenic mice that model the Tau pathology in AD and FTD^36^. What these data illustrate is the complexity in revealing the distribution of Tau, some of which may reflect nonspecific binding of the different anti-Tau antibodies that have been employed.

**Figure 2:**
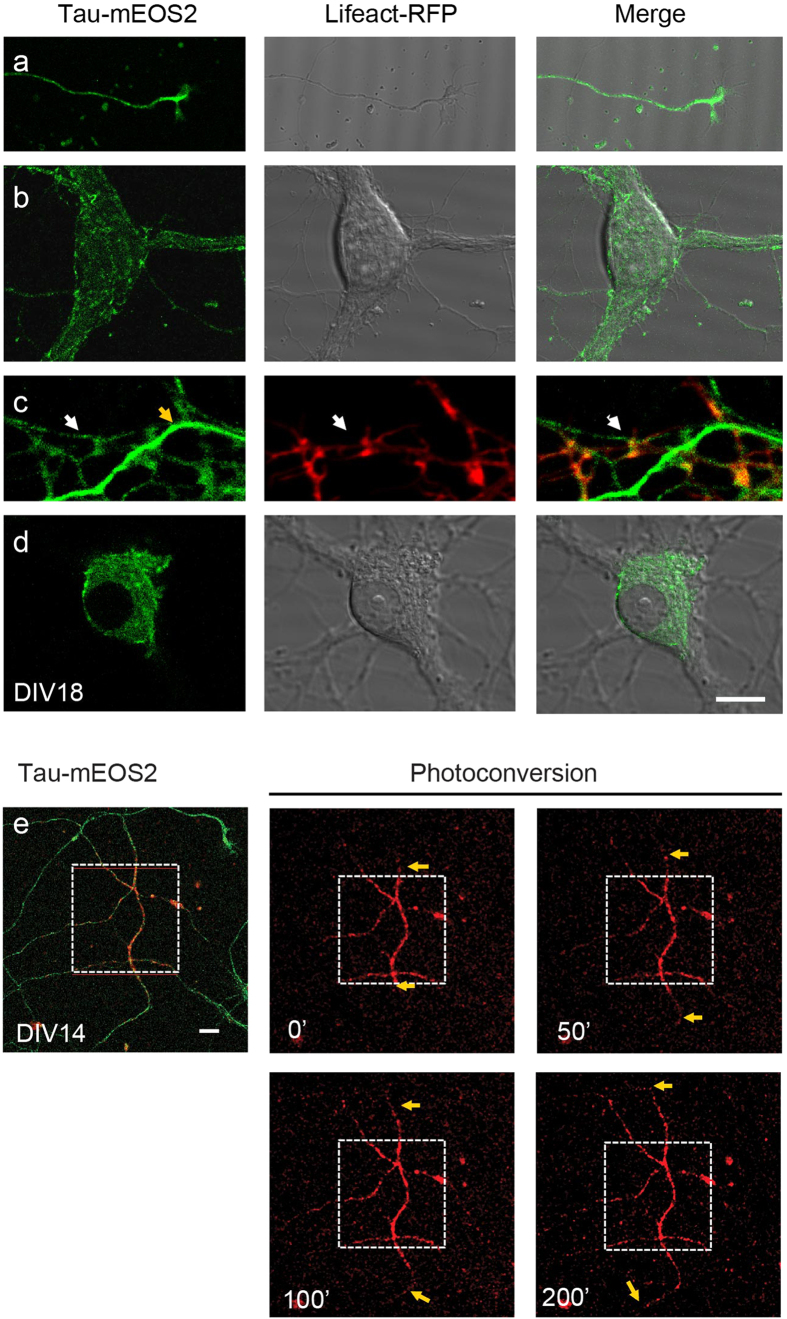
Tau-mEOS2 is localized to axons, dendrites and the soma but excluded from the nucleus. Mature DIV18 hippocampal cultures from Tau-mEOS2 mice cotransfected with the spine marker Lifeact-RFP to reveal dendritic spines. **(a)** Tau-mEOS2 mice show a strong axonal expression of EOS-tagged Tau, with a gradient towards the growth cone of distal axons. **(b)** Tau-mEOS2 is also present in the somatodendritic domain, (**c**) with very low levels in dendritic spines (zoom-in, white arrow: dendrite; yellow arrow: axon). (**d**) Within the limit of detection, EOS-tagged endogenous Tau cannot be visualized in the nucleus. **(e)** Time-lapse imaging of photo-converted mEOS2-tagged Tau reveals bidirectional transport of Tau in the axon at about ~0.25 μm/s. Yellow arrows indicate how far photo-converted Tau travelled. Scale bar: 10 μm (**a**–**d**) and 15 μm (**e**).

To clarify whether Tau is expressed at significant levels in dendrites and nuclei, we established primary hippocampal cultures from homozygous Tau-mEOS2 mice and used confocal imaging to visualize the mEOS2-tagged Tau in live neurons. We found that Tau-mEOS2 showed a strong axonal expression in mature (DIV18) neurons, with a gradient towards enrichment in the growth cone of distal axons (Fig. 2a). Tau-mEOS2 was further present in the somatodendritic domain (Fig. 2b), with very low levels in dendritic spines (Fig. 2c). Within the limit of detection, we did not visualize EOS-tagged endogenous Tau in the nucleus, suggesting that the level is either very low and/or that previously reported detection represented background reactivity (Fig. 2d). Interestingly, as for the staining of WT neurons with anti-Tau antibodies, Tau was found to be expressed throughout the cell, with an enrichment in the axon. This shows that, even with a reduced level of Tau, the neuron is capable of maintaining an axo-dendritic gradient. This is important for treatment strategies aimed at reducing Tau levels^37^.

To demonstrate that the endogenously expressed mEOS2-tagged Tau in the Tau-mEOS2 mice can be photo-converted, we assessed Tau transport in the axon. Time-lapse imaging revealed the bidirectional transport of Tau at about ~0.25 μm/s out of the region of interest (Fig. 2e). This value is lower than that previously obtained for over-expressed CFP-hTau40 in retinal ganglion cell neurons (0.4~0.6 μm/s) indicating that the transport of Tau may be affected by expression levels and the experimental system^38^.

**Figure 3:**
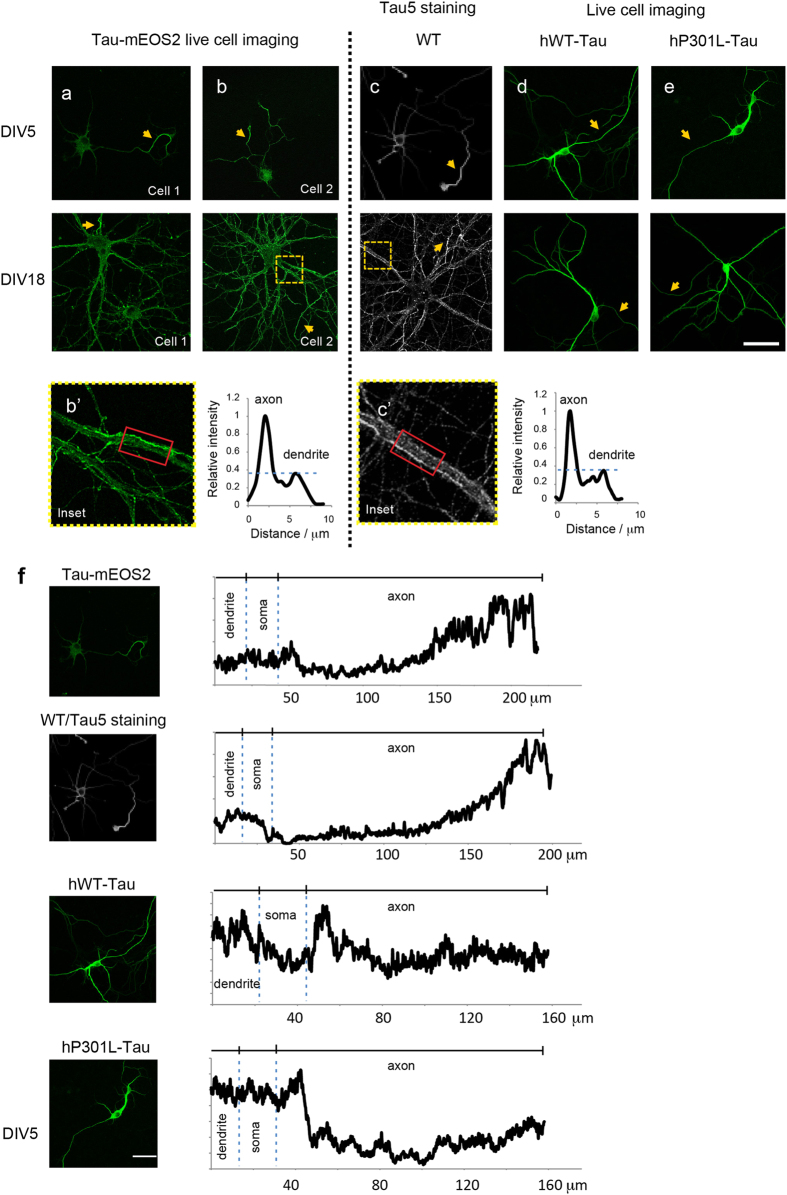
Tau in Tau-mEOS2 neurons shows an axo-dendritic distribution that differs from that of over-expressed Tau. Live cell imaging of Tau in Tau-mEOS2 hippocampal neurons (**a,b**) compared to neurons over-expressing EGFP-tagged hWT-Tau **(d)** and hP301L-Tau (**d,e)** at DIV5 and DIV18 in culture, and to WT neurons stained with the pan-tau-specific antibody Tau5 **(c)**. The yellow arrow indicates axons. Line profiles through an axon and a dendrite running in parallel displaying the relative intensity of Tau in these two compartments **(b’,c’)**. **(f)** Line profiles were established for the four experimental conditions to reveal the fluorescent Tau signal starting at a dendrite and then going through the soma and along the axon to its tip, demonstrating the altered axo-dendritic gradient in the over-expression systems. Scale bar: 50 μm.

We were next interested in determining whether the axo-dendritic gradient is impaired when Tau is over-expressed, as is routinely done to study Tau. For this, we used hippocampal cultures at two stages of maturation: DIV5, when neurons are immature, and DIV18, when the neurons have developed mature spines. We over-expressed EGFP-tagged forms of human WT (hWT-Tau) and human P301L mutant Tau (hP301L-Tau), one modelling the form of Tau that accumulates in AD and the other the form of Tau that accumulates in FTD. Importantly, as for the Tau-mEOS2 mice, the EGFP tag was added to the carboxy-terminus of the two over-expression constructs. However, the fact that an exogenous promoter rather than the endogenous promoter was used to drive expression of hWT and hP301L Tau could potentially result in a different spatial specificity, as it has been shown in transfection studies that the 3′ UTR of the Tau-encoding *MAPT* gene has a role in axonal targeting of the mRNA^39^. By DIV5, Tau was already found to be present in all major neuronal compartments (soma, axon and dendrite) in Tau-mEOS2 neurons (Fig. 3a,b). Moreover, it was axonally enriched, displaying a proximo-distal gradient, similar to that found in hippocampal cultures from WT C57Bl/6 mice that were stained with the anti-Tau antibody Tau5 and a fluorescent secondary antibody (Fig. 3c). By establishing line profiles through an axon and a dendrite running in parallel, the intensity of Tau in the axon was found to be approximately 2.5-fold higher than that in the dendrite both when Tau-mEOS2 cultures were captured and when WT cultures were stained with the Tau5 antibody (Fig. 3b’’). More generally, dendritic staining was found to be uniformly weak, whereas that in axons was generally more variable. Upon over-expression of either hTau-EGFP or hP301LTau-EGFP in WT neurons, both forms of Tau were found to accumulate throughout the neuronal cytoplasm, although the fluorescence intensity for the axon was much lower than that for the somatodendritic domain (Fig. 3d,e). This was already evident at DIV5 and was maintained with neuronal maturation (Fig. 3a–e, lower panel). We also established line profiles for all experimental conditions to reveal the fluorescent Tau signal starting at a dendrite and proceeding through the soma and along the axon to its tip. This not only demonstrated that the axo-dendritic gradient was inverted in the over-expression systems, but also revealed that the proximodistal gradient of Tau was abolished within the axon (Fig. 3f). Together these results demonstrate that, despite a 15-fold lower expression level, Tau-mEOS2 shows a distribution similar to that of endogenous Tau, which is distorted when Tau is over-expressed in its WT form or carrying a mutation (P301L) found in human tauopathy.

### Spine localization of over-expressed P301L Tau is increased compared with that of over-expressed WT Tau or endogenous mEOS2-tagged Tau

**Figure 4:**
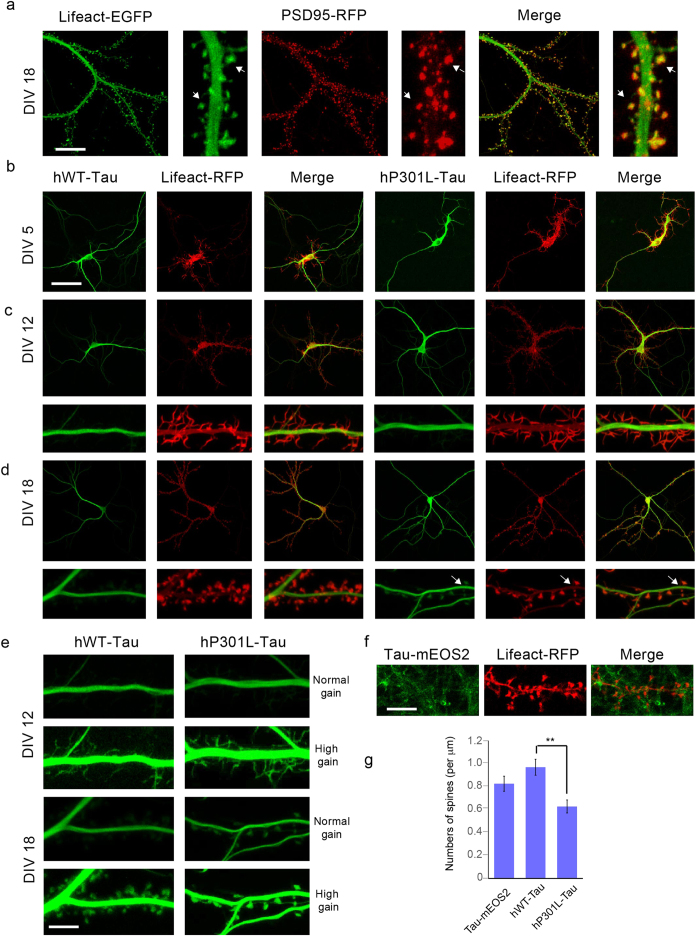
Increased spine localization of Tau and reduced spine numbers in neurons over-expressing P301L human Tau compared to WT human Tau over-expressing and Tau-mEOS2 neurons. (**a**) Filamentous (F-) actin visualized with Lifeact-EGFP co-localizes with PSD-95 in dendritic spines (white arrows in cropped, zoomed-in images indicate spines). (**b–d**) Live-cell imaging of hWT-Tau and hP301L-Tau over-expressing neurons at three stages of neuronal maturation, DIV5 (**b**), 12 (**c**) and 18 (**d**), (plus zoom-ins) reveals that both forms of Tau are mainly excluded from filopodia. Spine localization of hP301L-Tau is more pronounced than that of hWT-Tau (**b–d**), which only becomes visible by increasing the gain (**e**). Endogenous Tau (Tau-mEOS2) is also targeted to spines, albeit at much lower levels (**f**,**g**). Spine counts do not differ between Tau-mEOS2 and hWT-Tau over-expressing neurons, but are massively reduced in hP301L-Tau over-expressing neurons (two-tailed unpaired Students t-test, **P < 0.01). Scale bar: 20 μm (**a**), 50 μm (**b–d**), 10 μm (**e,f**).

Tau localization in the dendrite has previously been found to extend to spines; however, levels of endogenous Tau were reported to be low, unless Tau was over-expressed, causing it to enter spines in a phosphorylation-dependent manner^25^,^40^. To assess spine localization of Tau, we co-expressed EGFP-tagged Lifeact to visualize F-actin^41^, as a spine marker, due to its co-localization with RFP-tagged PSD95 in DIV18 WT neurons (Fig. 4a).

Using live-cell imaging to assess hWT-Tau and hP301L-Tau over-expressing neurons at three stages of neuronal maturation (DIV5, 12 and 18), we found that although both forms of Tau were mainly excluded from filopodia within the limit of detection, they were targeted to dendritic spines, with a lower intensity of spinal compared to dendritic Tau, and a more pronounced spine localization of P301L Tau compared to WT human Tau (Fig. 4b–d), as shown by applying a higher gain to expose spine localization (Fig. 4e). Endogenous Tau was also targeted to spines, albeit at much lower levels (Fig. 4f). Unambiguously identifying spine localization is easier in hWT-Tau and hP301L-Tau over-expressing neurons than in Tau-mEOS2 cultures, as in the latter only a fraction of neurons is fluorescently tagged, allowing a better discrimination against a background of non-fluorescing neurons. We next performed spine counts and found that these did not statistically differ between hTau-mEOS2 and hWT-Tau over-expressing neurons, but were significantly reduced in hP301L-Tau over-expressing neurons (Fig. 4g). Hoover and colleagues previously reported no reductions in their P301L-Tau cultures, and whereas we observed approximately 95 spines/100 μm in WT cultures, they reported around 40 spines/100 μm. However, the two studies differ in several aspects: they transfected 0N4R and not 2N4R Tau, and they transfected at DIV7-10 and analysed the neurons at DIV21^25^,^40^, whereas we transfected the neurons only at DIV18 and analysed them at DIV19/20. Together, our findings reveal that even under physiological conditions, Tau is targeted to dendritic spines, and that over-expression of P301L mutant Tau causes a decrease in spine density.

### FRAP analysis reveals a similar mobility of Tau in dendrites and axons, but in both compartments, Tau-mEOS2 is less mobile than hWT-Tau and hP301L-Tau

We next used the FRAP assay to address the dynamics of Tau in DIV18-21 hippocampal cultures under over-expression conditions compared to the lower expression encountered in the Tau-mEOS2 mice, and analysed the recovery profiles to determine the mobility and mobile fraction in the different neuronal compartments. It has to be considered that trafficking of molecules into the spine is unidirectional (from the dendritic shaft through the spine neck into the spine head), whereas in the axon and dendrite, Tau can move into the bleached area from both ends. The bleached spine is also much smaller than the bleached area of either the axon or dendrite, which generally results in a faster recovery. Therefore, the spine FRAP data were anaysed separately from those obtained for axons and dendrites.

**Figure 5:**
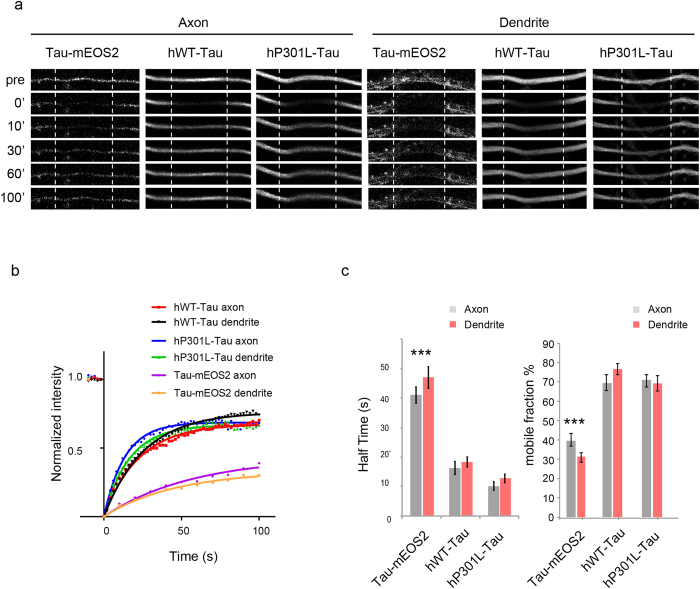
FRAP analysis in axons and dendrites of P301L and WT human tau over-expressing and Tau-mEOS2 neurons. **(a)** Grey-scale images of axons and dendrites from Tau-mEOS2 neurons and hP301L and hWT Tau over-expressing neurons showing time-points before (pre) and after bleaching (t = 0, 10, 30, 60 and 100 s during fluorescence recovery). FRAP regions (100 μm) are indicated by dashed lines. **(b)** Recovery profiles and **(c)** FRAP analysis of Tau-mEOS2 compared to over-expressed hWT- and hP301L-Tau in the dendrite and axon. In both compartments, the t^1/2^ of hTau-mEOS2 is higher than for either hWT-Tau or hP301L-Tau, and the mobile fraction is much smaller. (One-way ANOVA, ***P < 0.005; N > 15).

We first determined the dynamics of hWT-Tau, hP301L-Tau and Tau-mEOS2 in the axon and dendrite (Fig. 5a,b), and determined the mobility by calculating the diffusion constant t^1/2^ (half time, the time required for the fluorescence to reach 50% recovery, with more mobile molecules displaying a smaller t^1/2^) and the mobile fraction (Fig. 5c). We found in both the dendrite and the axon that the mobility of Tau-mEOS2 was lower than that of either hWT-Tau or hP301L-Tau. We further found when either hWT or hP301L Tau was over-expressed, that the mobile fraction of Tau was between 70 and 80% of the total pool, whereas for Tau-mEOS2, this fraction was reduced by a factor of two, possibly reflecting less efficient binding of Tau to microtubules when Tau is over-expressed. Interestingly, for any experimental condition, we did not observe a difference in the mobility or mobile fraction of Tau between axons and dendrites.

**Figure 6:**
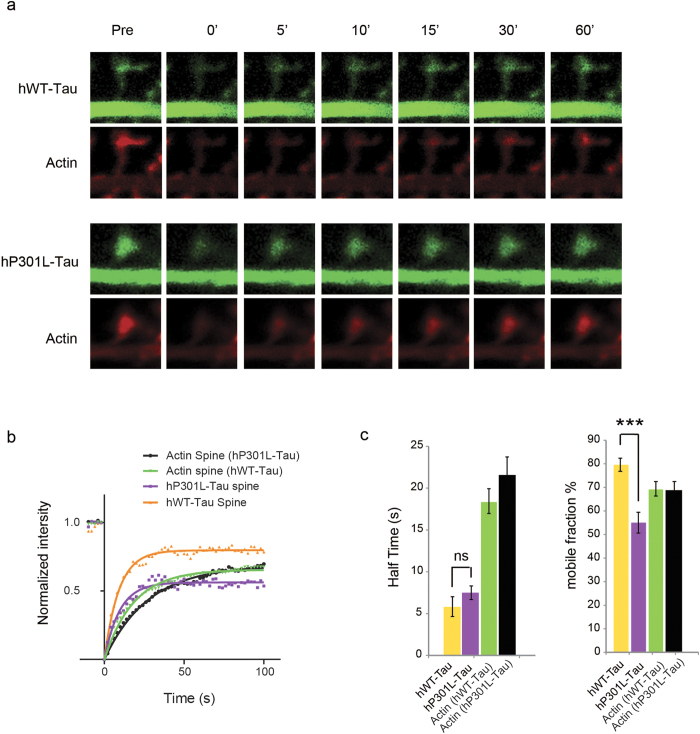
Tau and F-actin dynamics in spines. **(a)** Representative images of hP301L and hWT Tau over-expressing neurons showing time-points before (pre) and after bleaching (t = 0, 5, 10, 15, 30 and 60 s during fluorescence recovery). **(b)** Recovery profiles and **(c)** FRAP analysis of hWT-Tau and hP301L-Tau together with the corresponding F-actin in spines. F-actin is less mobile than either form of Tau, and the mobility and recovery of actin are not affected by the form of Tau being over-expressed. There is a smaller mobile fraction in hP301L-Tau compared to hWT-Tau neurons (two-tailed unpaired Student’s t-test, ***P < 0.005; N > 20).

We next determined the dynamics of hWT-Tau and hP301L-Tau in the spine (the Tau-mEOS2 signal was too weak to be included in the analysis) (Fig. 6a,b), and determined the diffusion constant t^1/2^ and the mobile fraction in this subcellular compartment (Fig. 6c). Because F-actin is a critical Tau-interacting protein and is enriched in spines, we specifically determined the mobility of F-actin (Lifeact-RFP) depending on the form of Tau being expressed. This revealed that although F-actin was less mobile than tau in spines, its dynamics was not affected by the form of Tau being over-expressed (which was also the case for dendrites and axons). Interestingly, there was a smaller mobile fraction of hP301L-Tau in the spines (55%) compared to that of hWT-Tau (80%). One explanation may be that more hP301L-Tau is tethered with F-actin in spines compared to hWT-Tau, which reduces its turnover rate in spines. We also found that whereas the mobility of hP301L-Tau in the axon and dendrite was higher than that of hWT-Tau (see above), it did not differ significantly in the spine.

### Conclusions and outlook

In our study, we present the Tau-mEOS2 mouse strain as an interesting system in which to investigate transport in neurons, under conditions of both physiologically and pathologically phosphorylated Tau. Regarding the significantly reduced Tau levels in Tau-mEOS2 compared with WT mice it remains to be seen whether these can be increased by generating mice in which the mEOS2 tag is added to the amino-terminus of Tau. In the Tau-mEOS2 mice described above, we observed a Tau gradient that differed from that of over-expression systems. A possible explanation is provided by a recent study that used photo-conversion of human WT Tau tagged with photo-convertible Dendra2 followed by over-expression in relatively young cortical cultures to reveal an anterograde flow across the axon initial segment into the axon, but no flow back into the somato-dendritic compartment, explaining axonal enrichment of Tau^29^. When Tau was hyperphosphorylated using the phosphatase inhibitor okadaic acid, the retrograde barrier was found to break down functionally, with Tau now flowing into the somato-dendritic domain where it is known to accumulate in AD^29^. Because okadaic acid can have multiple effects that go beyond Tau hyperphosphorylation, the study further used a ‘4KXGE’ Tau mutant mimicking constitutive phosphorylation, which again showed a functional break-down of the retrograde barrier and a relocalization of this form of Tau into the somatodendritic domain. This could potentially explain why over-expressed Tau accumulates in the somatodendritic domain, whereas endogenous Tau is more localized to the axon. However, there are also alternative explanations, as the over-expression of Tau could lead to a saturation effect, increasing the pool of Tau that is not captured by microtubules and hence starts accumulating in the somatodendritic domain.

In conclusion, with the Tau-mEOS2 mice we have developed a versatile tool that will allow for the monitoring of endogenous Tau in response to a range of stimuli. For example, neuronal activity has been found to drive physiological levels of Tau into the spine in a process in an incompletely understood process that is impaired by by the peptide Aβ that drives AD^24^. Here, the Tau-mEOS2 mice may be a tool to understand this process in more detail, using a system that avoids over-expression.

## Materials and Methods

### Ethics statement

All experimental procedures in this study were conducted under the guidelines of the Australian Code of Practice for the Care and Use of Animals for Experimental Purposes and were approved by the University of Queensland Animal Ethics Committee (QBI/412/14/NHMRC; QBI/027/12/NHMRC). Mice were maintained on a 12-hour light/dark cycle and housed in a PC2 facility with *ad libitum* access to food and water.

### Generation of Tau-mEOS2 mice using TALEN gene editing

Mice with an EOS cassette inserted into the carboxy-terminus of the open reading frame of the tau-encoding *MAPT* gene (Tau-mEOS2 mice) were generated using the TALEN gene editing tool^30^. Three TALEN pairs that target a sequence near the stop codon of exon 12 of *MAPT* were designed using the online tool “TAL Effector Nucleotide Targeter 2.0” (https://tale-nt.cac.cornell.edu/node/add/talen). Three TALEN pairs that were predicted to yield a high targeting efficiency and low off-target effects were selected and assembled using the TALEN toolkit (Addgene Kit # 1000000019) and the PCR-golden gate ligation method^42^. A donor construct comprising the mEOS2 cassette flanked by 800 bp long arms both upstream and downstream of the genomic sequence containing the *MAPT* stop codon was PCR-amplified and subcloned into the pGEM-T easy vector (Promega) using the NEB Gibson assembly kit. TALEN activity was evaluated in N2A cells using the T7E1 assay. The pair that gave the highest activity was then used for *in vitro* transcription, using the Ambion megascript T7 kit. TALEN mRNAs (10 ng/μl each) were combined with the donor construct (15ng/μl) and injected into pronuclei of C57BL/6 single cell mouse embryos followed by transfer into pseudo-pregnant foster mice.

The following primer pairs were used to identify offspring (F0) animals with a correct in-frame mEOS2 targeting event.

Primer P1 (forward): 5′-CTATCTCTGACTCATTCTGTCAAATCTT-3′,

Primer P2 (reverse): 5′-GAAACGACTGCTTAAAATAGTCTTGTAT-3′,

Primer P3 (forward): 5′-CGTAAACGGGCACCACTTTGTGATCGACGG-3′, and

Primer P4 (reverse): 5′-GATACAGTGGCTGTGTGAGGGATGGGAGGC-3′.

The PCR products obtained with primers pairs P1/P2 and P3/P4 were gel-purified and subcloned into the pGEM-T easy vector for sequence verification using the following primers:

T7 : 5′-TAATACGACTCACTATAGGG-3′, and

SP6: 5′-ATTTAGGTGACACTATAG-3′.

### Real-time quantitative PCR analysis

For real-time quantitative PCR (RT-qPCR), left brain hemispheres were dissected and homogenized in TRIzol buffer (Thermo Fisher Ambion) for mRNA extraction. A reverse transcription kit (Thermo Fisher Invitrogen) was used to synthesize cDNA, followed by incubating the cDNA with a SYBR Green master mix (Bio-Rad) to perform an RT-qPCR reaction (C1000 Touch Thermo Cycler operating with the CFX384 Optical Reaction Module).

The following primers used were:

Mouse *Mapt* forward, 5′-GACCTAAAGAATGTCAGGTCG-3′;

Mouse *Mapt* backward, 5′-GACGTGTTTGATATTATCCTT-3′;

Mouse *Gapdh* forward, 5′-AACTTTGGCATTGTGGAAGG-3′

Mouse *Gapdh* backward, 5′-GGATGCAGGGATGATGTTCT-3′

### Primary hippocampal cultures

Embryonic day (E)17 or E18 hippocampal neurons were obtained from WT, Tau knock-out (KO)^32^, and Tau-mEOS2 mice and plated onto poly-L-lysine coated coverslips in a 12-well plate at a density of 80,000 cells/well^43^. For their culture, Neurobasal medium (Gibco) was supplemented with 5% foetal bovine serum (Hyclone), 2% B27, 2 mM Glutamax (Gibco) and 50 U/ml penicillin/streptomycin (Invitrogen). The medium was changed to serum-free Neurobasal medium 24 h post-seeding and half the medium was changed twice a week. For live-cell confocal imaging, 200,000 cells were plated into 35 mm glass-bottom dishes (*In Vitro* Scientific) using the above medium, which was replaced by phenol red-free Neurobasal medium 24 h later.

### Western blot analysis and immunocytochemistry

For western blot analysis and immunocytochemistry, two Tau-specific antibodies were used: total Tau (Dako, polyclonal, 1:5,000), and Tau5 (Thermo Fisher, monoclonal, 1:5,000). Both IRDye 800CW goat anti-rabbit IgG and IRDye 680RD goat anti-mouse IgG (Licor) were used as secondary antibodies for western blot analysis. For immunocytochemistry in combination with Tau5, Alexa Fluor 555-labelled goat-anti-mouse antibody (LifeTechnologies, Thermo Fisher, 1:500) was used for detection. To detect actin, phalloidin-Alexa Fluor 647 (New England Biolabs, 1:100) was used.

### Live cell imaging, FRAP assay and photo-conversion

Primary neurons were transfected using Lipofectamine 2000 and used for live-cell imaging 24 to 48 h post-transfection. The following constructs were used for transfection: hTau-EGFP (human full-length 2N4R Tau with carboxy-terminally tagged EGFP), hP301L-EGFP (FTD P301L mutant 2N4R Tau with carboxy-terminally tagged EGFP), Lifeact-RFP (pmTagRFP-T-Lifeact-7, Addgene Plasmid #54586), and Lifeact-EGFP (kindly provided by Dr. Fred Meunier). Photo-conversion was achieved using the 405 nm UV laser on the LSM710 confocal microscope (Zeiss). For the FRAP assay, DIV 18–21-transfected neurons were analysed using a Zeiss LSM 710 confocal microscope equipped with a 5% CO_2_, 37 °C humidified cage. Time-lapse images of the GFP and RFP channels were captured with a Zeiss Plan-Apochromat 63x Oil DIC (N.A. = 1.4) objective at 2 s intervals using a 4× optical zoom. Regions of interest (ROIs) of the same size or length in the spine, axon or dendrite were photo-bleached by scanning with a 405 nm and 488 nm laser, respectively, combined at the maximum power, at a scanning speed of 3.5 μs/pixel which was repeated five times. Images were analysed using Image J and Prism 6. For each ROI, the total intensity was measured and background intensities were subtracted. A dendritic or axonal region away from the FRAP ROIs was used to compensate for the fluorescence loss during image acquisition. Data from different cells were normalized to pre-bleaching as 100% and immediately post-bleaching as 0%. The following formula was used for the fitting of FRAP curves: % Recovery = (1 − e^−Kt^) ∙ 100%, with K being the recovery rate constant, and (t1/2) the half-time = ln(2)/k (half-time being the time required for fluorescence to reach 50% recovery). Data are presented throughout as mean ± SEM.

## Additional Information

**How to cite this article**: Xia, D. *et al*. Mobility and subcellular localization of endogenous, gene-edited Tau differs from that of over-expressed human wild-type and P301L mutant Tau. *Sci. Rep.* **6**, 29074; doi: 10.1038/srep29074 (2016).
